# Comparison of aspiration with sclerotherapy and laparoscopic deroofing for the treatment of symptomatic simple renal cysts: a systematic review and meta-analysis

**DOI:** 10.1007/s13304-021-01042-2

**Published:** 2021-04-01

**Authors:** Andrea Maugeri, Gabriele Fanciulli, Martina Barchitta, Antonella Agodi, Guido Basile

**Affiliations:** 1 Department of Medical and Surgical Sciences and Advanced Technologies “GF Ingrassia”, University of Catania, Via S. Sofia 87, 95123 Catania, Italy; 2 Department of General Surgery and Medical-Surgical Specialties, University of Catania, via S. Sofia, 78, 95123 Catania, Italy

**Keywords:** Renal cysts, Percutaneous aspiration, Sclerotherapy, Laparoscopic deroofing, Marsupialization

## Abstract

There are currently several strategies for the treatment of symptomatic simple renal cysts, such as aspiration with sclerosants and laparoscopic deroofing. However, no clear indication exists for choosing between them. Here, we carried out a systematic review and a meta-analysis of studies, which compared symptomatic and radiological success between aspiration with sclerotherapy and laparoscopic deroofing. Results were reported as relative risk (RR) and 95% confidence interval (95% CI) using laparoscopic deroofing as control group. The symptomatic and radiological successes were evaluated by 6 and 3 studies, respectively. Notably, aspiration with sclerotherapy was associated with higher risk of failure than laparoscopic deroofing (RR = 2.82; 95% CI = 1.84–4.31 for symptomatic failure; RR = 8.31; 95% CI = 4.22–16.38 for radiological failure). On the other hand, however, aspiration with sclerotherapy was associated with less frequent complications, shorter treatment duration and post-treatment hospital stay, and lower costs. Thus, our work underlines benefits and drawbacks of each intervention, raising the need for further studies to design guidelines for the management of simple renal cysts.

**Supplementary Information:**

The online version contains supplementary material available at 10.1007/s13304-021-01042-2.

## Introduction

Renal cysts are very common lesions with a prevalence ranging from 5 to 20% in the general population and especially in the elderly [1]. According to their size, small renal cysts (diameter < 4 cm) are generally asymptomatic and only occasionally diagnosed by ultrasonography, computed tomography, or magnetic resonance imaging. By contrast, renal cysts with medium (> 4 cm) or large diameter (> 7 cm) are more likely to become symptomatic, with several complications including back pain, urinary tract infection, hemorrhage, hematuria, and hypertension [2–4]. However, there is not a direct association between cyst’s size and symptoms, because even a small cyst can lead to a clinical manifestation depending on its location [5]. The Bosniak classification—proposed for the first time in 1986—allows to classify renal cysts in five categories (i.e., I, II, IIF, III, and IV) based on their morphology and enhancement properties [6]. Specifically, type I and II categories include simple cysts with low likelihood of becoming symptomatic and malignant. By contrast, complex renal cysts require follow-up (type IIF) or immediately surgery (types III and IV) to avoid malignancy. Yet, it has been estimated that 8% of simple renal cysts become symptomatic and need intervention [7, 8]. Due to their growth, indeed, simple renal cysts can cause symptoms such as hypertension, hematuria, pain, parapelvic obstruction, and, in some cases, can undergo rupture [5]. For this reason, an intervention for this disease is indicated for all symptomatic patients, with the main goal of excising the cyst wall and evacuating the fluid [1, 6, 9]. There are currently several strategies for the treatment of symptomatic simple renal cysts, such as percutaneous aspiration with or without sclerosing agents, laparoscopic deroofing with transperitoneal and retroperitoneal approaches, robotic surgery, and open surgery [5]. Although the first two techniques are the most commonly used [5], to our knowledge, no clear indication exists for choosing between aspiration with sclerotherapy and laparoscopic deroofing. To address this issue, we carried out a systematic review and a meta-analysis of studies, which evaluated and compared the efficacy of aspiration with sclerotherapy and laparoscopic deroofing for the treatment of symptomatic simple renal cysts.

## Methods

### Literature search and selection criteria

The methodology of the current systematic review was in accordance with the Preferred Reporting Items for Systematic Reviews and Meta-analyses (PRISMA) statements and the Cochrane Handbook’s guidelines [10] (Supplementary Table 1). Two of the authors (AM and GF) carried out a literature search of articles indexed in the PubMed and Web of Sciences databases from January 1985 to February 2021, using the following keywords: ((renal cysts) OR (renal cyst)) AND ((sclerotherapy) OR (aspiration)) AND ((laparoscopic decortication) OR (laparoscopic deroofing) OR (marsupialization)). Duplicates were manually identified and excluded by the two authors. The following inclusion criteria had to be meet: (i) prospective or retrospective studies; (ii) on patients with symptomatic simple renal cysts (Bosniak categories I and II); (iii) which compared aspiration with sclerotherapy and laparoscopic deroofing; (iv) in terms of radiological and/or symptomatic success. By contrast, the following documents were excluded: (i) studies on patients with other kidney diseases (e.g., polycystic kidney); (ii) studies recruiting patients < 18 years old; or (iii) not comparing the above-mentioned techniques; (iv) letters, case reports and case series, reviews, non-English articles, and abstracts without full text. Titles and abstracts of all identified articles were independently screened by two authors (AM and GF), who applied inclusion/exclusion criteria. All articles deemed potentially eligible were full-text reviewed to assess whether eligibility criteria were fully met. Controversies were resolved by consensus or consultation with a third author (AA).

### Data extraction

Two of the authors (AM an GF) extracted the following information from all the included studies: first author, year of publication, country, study design, follow-up duration, sample size, age, proportion of men, type of cysts, diameter of cysts, type of interventions, radiological success, and symptomatic success. In addition, when available, the authors collected information on the rate of complications (according to the Clavien–Dindo Grading System or not), treatment duration and post-treatment hospital stay. Controversies were resolved by consensus or consultation with a third author (AA).

### Statistical analysis

The primary outcome of the present study was the success of treatment, examined through radiological and symptomatic assessment. Secondary outcomes included the rate of complications, treatment duration, and post-treatment hospital stay. For the primary outcomes, the authors calculated and compared the proportions of success and failure between aspiration with sclerotherapy and laparoscopic deroofing. The meta-analyzed effect sizes were reported as the relative risk (RR) and 95% confidence interval (95% CI) of treatment failure in the aspiration with sclerotherapy group compared with laparoscopic deroofing. Heterogeneity across studies was tested using the *Q*-statistics and the I2 index, and a fixed effect model was applied in absence of significant heterogeneity. Instead, in presence of significant heterogeneity across studies (*Q*-statistics < 0.1 and *I*^2^ > 50%), a random effect model using the reduced maximum-likelihood (REML) approach was applied.

### Risk of bias and quality assessment

The extent of publication bias was explored by funnel plots and tested using Egger’s test. Moreover, two of the authors (AM and GF) used the ROBINS-I tool for assessing the risk of bias in non-randomized studies [11]. Specifically, the tool allowed to assess the following domains of bias: bias due to confounding; bias in selection of participants into the study; bias in classification of interventions; bias due to deviations from intended interventions; bias due to missing data; bias in measurement of outcomes; bias in selection of the reported result. Finally, the level of evidence was assessed according to criteria from the Oxford Centre for Evidence-Based Medicine (OCEBM) [12].

## Results

### Selection and characteristics of included studies

**Fig. 1:**
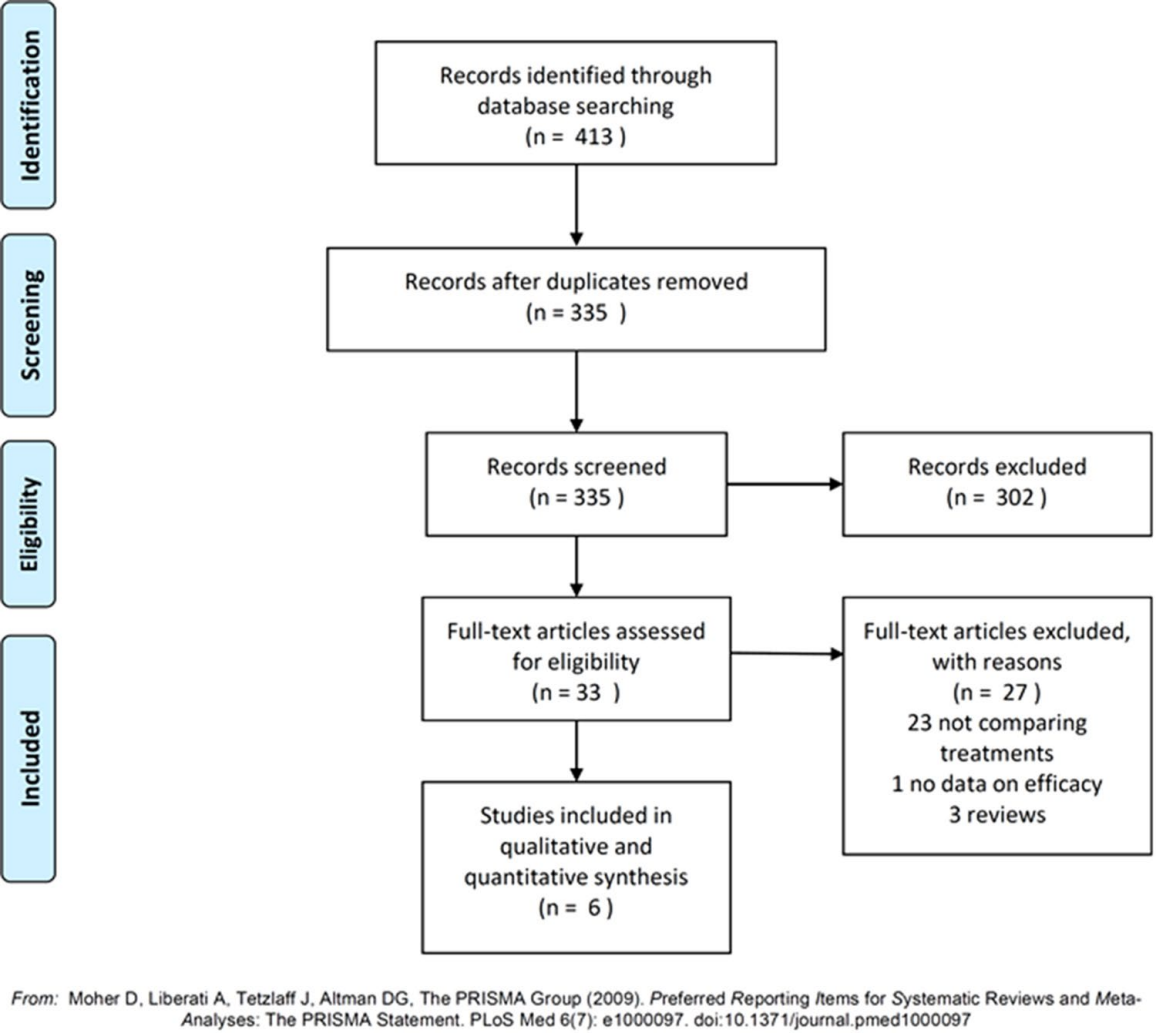
PRISMA flow diagram of study selection

The PRISMA flow diagram describing the study selection is reported in Fig. 1. Literature search identified a total of 413, of which 335 were screened once duplicates had been removed. After full-text screening of 33 articles, 23 articles not comparing aspiration with sclerotherapy and laparoscopic deroofing, 1 study not reporting success rates, and 3 reviews were excluded. Thus, a total of 6 articles were included in the present systematic review and meta-analysis (Table 1). In general, included articles were published from 2003 to 2020 and described studies conducted in India [13], Turkey [4, 14–16], South Korea [1], United Kingdom [17], and China [18]. The only randomized study was that by Agarwal et al. [13], which consisted in two parallel arms comparing aspiration with 1% polidocanol sclerotherapy and retroperitoneal laparoscopic deroofing. The remaining 5, instead, were non-randomized studies with a prospective [18] or retrospective [15–18] data collection. The latter compared aspiration and 95% ethanol sclerotherapy with laparoscopic deroofing based on the retroperitoneal or the transperitoneal approach. More details on techniques used in both arms are provided in Table 2. The follow-up duration varied from 6 to 35 months, and the overall sample size ranged from 13 to 1194 participants. All patients were from 18 to 87 years of age and almost all studies included a higher proportion of men. The diameter of cysts ranged from 3 to 16 cm, and all studies evaluated symptomatic cysts of Bosniak category I and/or II, except of Okeke and colleagues [17] that did not define cyst’s classification.

**Table 1:** Characteristics of studies included in the systematic review

First author and publication year	Study design	Follow-up (months)	Country	Sample size (AS/LD)	Age ( years)	Gender	Type of cysts	Cyst diameter (cm)
Agarwal et al. 2012	Prospective randomized study	12	India	40 (20/20)	25–68	55% men	Symptomatic cysts (Bosniak category I)	5.2–8.6
Bas et al. 2015	Retrospective study	35	Turkey	184 (149/35)	18–80	57.1% men	Symptomatic cysts (Bosniak category I–II)	3–16
Choi et al. 2020	Prospective study	6	South Korea	80 (40/40)	43–87	66.3% men	Symptomatic cysts (Bosniak category I)	5.33–11.8
Efesoy et al. 2015	Retrospective study	6	Turkey	80 (42/38)	21–74	57.9% men	Symptomatic cysts (Bosniak category I)	8.5 ± 2.7
Okeke et al. 2003	Retrospective study	12	UK	13 (7/6)	25–84	30.8% men	Symptomatic cysts	4.5–16
Shao et al. 2013	Retrospective study	12	China	1194 (208/986)	34–76	53.8% men	Symptomatic cysts (Bosniak category I–II)	5–15.1

*AS* aspiration with sclerotherapy, *LD* laparoscopic deroofing, *UK* United Kingdom

**Table 2:** Details on treatments for each study included in the systematic review

First author and publication year	Laparoscopic deroofing	Percutaneous aspiration
Agarwal et al. 2012	Retroperitoneal approach with three ports and patient in the flank position	The cyst was punctured and aspirated using an 18-gauge needle, under the guidance of ultrasonography with the patient in a prone position. After aspiration, 1% polidocanol in a volume equivalent to 10% of cyst volume was instilled. No attempt was made to aspirate or drain the sclerosant after instillation
Bas et al. 2015	Transperitoneal approach with three ports at a 45° flank position for anteriorly located renal cysts and the retroperitoneal approach with three ports at a flank position for posteriorly and laterally located cysts	The cyst was punctured and aspirated using an 18-gauge needle, under the guidance of ultrasonography with the patient in a prone position. After aspiration, 95% ethanol in a volume equivalent to 25% of cyst volume was instilled. A catheter was then clamped for 20 min while the patient was asked to move into different positions to help distribute the ethanol over the cyst wall; the catheter was then opened and drained completely by aspiration. The number of sessions depended on the cyst volume
Choi et al. 2020	Transperitoneal approach with three ports at a 45° flank position for anteriorly located renal cysts and the retroperitoneal approach with three ports at a flank position for posteriorly and laterally located cysts	The cyst was punctured and aspirated using an 18-gauge needle, under the guidance of ultrasonography with the patient in a lateral decubitus position. After aspiration, 95% ethanol in a volume equivalent to 25% of cyst volume was instilled. Subsequently, the position of the patient was changed every 5 min to ensure contact of ethanol with the entire inner surface of the cyst. The ethanol was then re-aspirated completely
Efesoy et al. 2015	Transperitoneal approach with three ports and patient in the flank position	The cyst was punctured and aspirated using an 18-gauge needle, under the guidance of ultrasonography with the patient in a lateral decubitus position. After aspiration, 95% ethanol in a volume equivalent to 25% of cyst volume was instilled. Subsequently, the position of the patient was changed every 5 min to ensure contact of ethanol with the entire inner surface of the cyst. The ethanol was then re-aspirated completely
Okeke et al. 2003	Transperitoneal approach with three ports and patient in the flank position	The cyst was punctured and aspirated under the guidance of ultrasonography. After aspiration, 95% ethanol in a volume equivalent to 20% of cyst volume was instilled. Subsequently, the position of the patient was changed every 5 min to ensure contact of ethanol with the entire inner surface of the cyst. The ethanol was then re-aspirated completely
Shao et al. 2013	Retroperitoneal approach with three ports and patient in the flank position	The cyst was punctured and aspirated using an 18-gauge needle, under the guidance of ultrasonography. After aspiration, 95% ethanol in a volume equivalent to 20% of cyst volume was instilled

### Symptomatic and radiological success of interventions

**Fig. 2:**
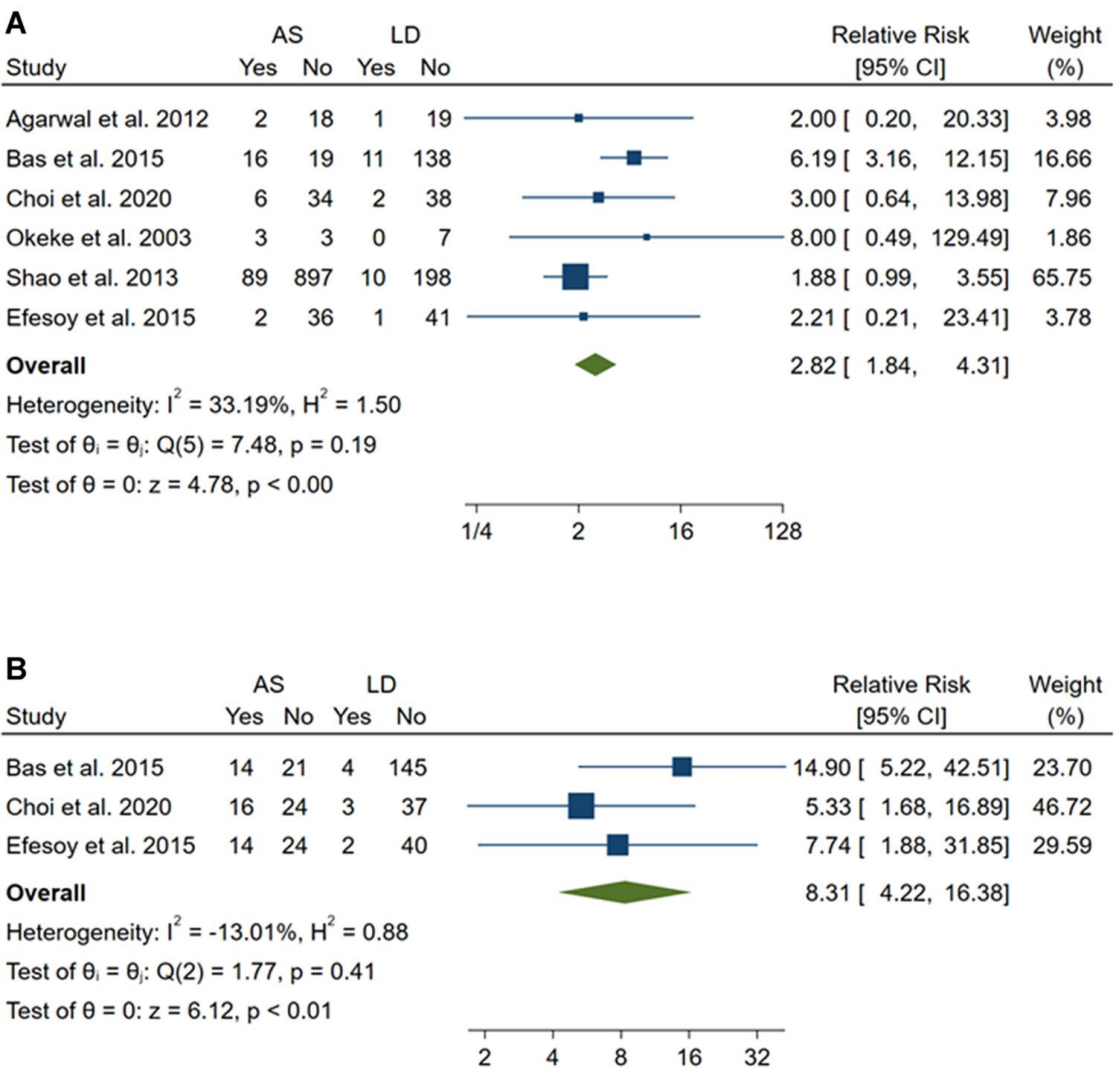
Forest plots of comparison between aspiration with sclerotherapy (AS) and laparoscopic deroofing (LD) in terms of symptomatic (**a**) and radiological (**b**) failure. Effect sizes are expressed as the relative risk and 95% confidence interval (95% CI) of AS using LD as control group. Pooled effect sizes were obtained through the fixed effect model

**Fig. 3:**
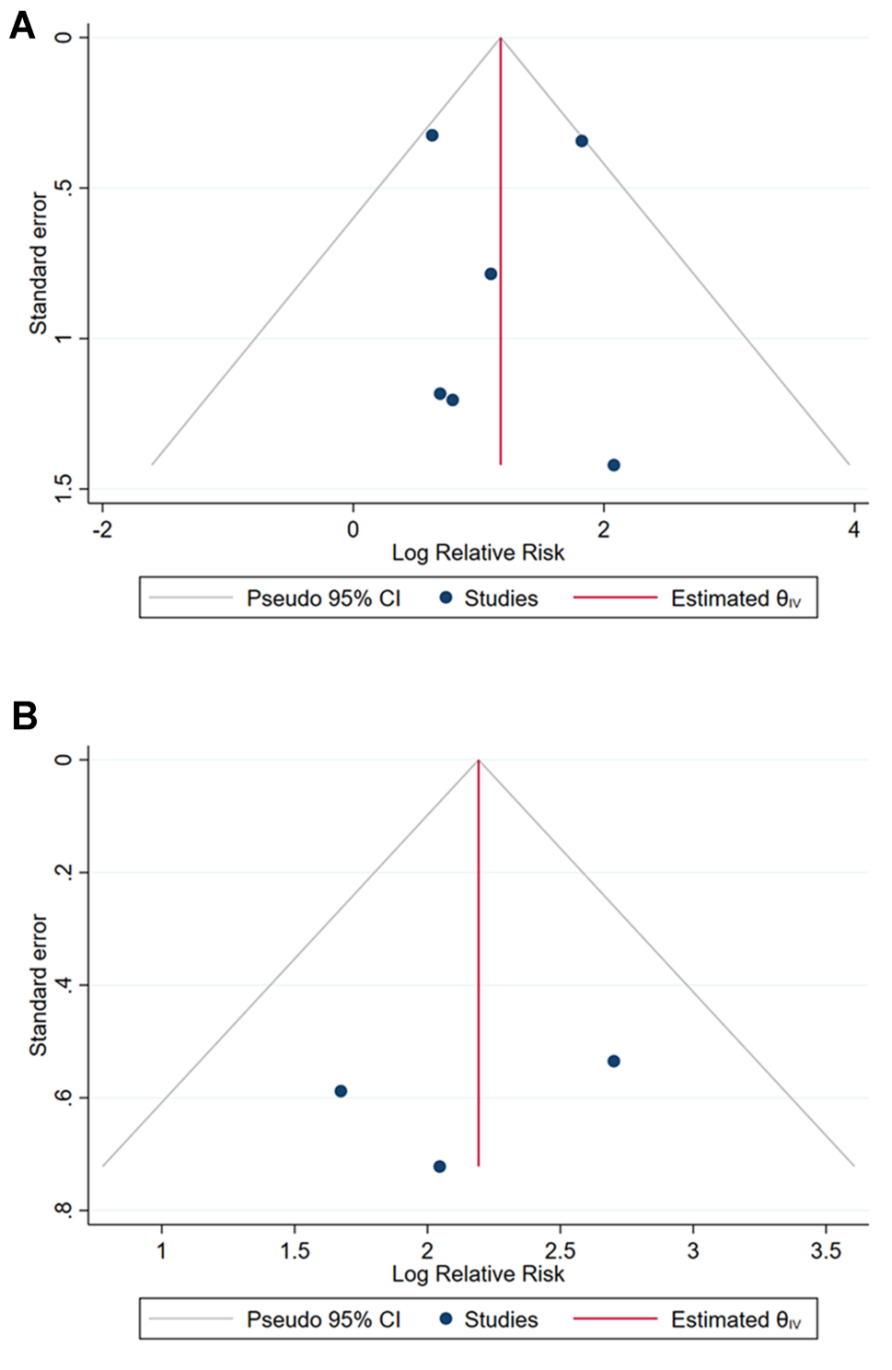
Funnel plots of comparison between aspiration with sclerotherapy and laparoscopic deroofing in terms of symptomatic (**a**) and radiological (**b**) failure

Overall, the present systematic review included 1591 patients who underwent aspiration with sclerotherapy (*n* = 1125) or laparoscopic deroofing (*n* = 466) (Table 3). The symptomatic success was evaluated by 6 studies [1, 13, 15–18] and ranged from 50 to 94.7% for the aspiration with sclerotherapy and from 92.6 to 100% for the laparoscopic deroofing. Similarly, the radiological success was evaluated by 3 studies [1, 15, 16], and ranged from 60 to 63.2% for the aspiration with sclerotherapy and from 92.5 to 97.5% for the laparoscopic deroofing. In line with these findings, the meta-analyses showed that aspiration with sclerotherapy was associated with higher risk of symptomatic (RR = 2.82; 95% CI = 1.84–4.31) and radiological (RR = 8.31; 95% CI = 4.22–16.38) failure if compared with laparoscopic deroofing (Fig. 2). The inspection of Funnel plots revealed a nearly symmetrical shape for each outcome and the Egger’s test confirmed no significant publication bias (*p* = 0.811 for symptomatic success and *p* = 0.171 for radiological success; Fig. 3).

**Table 3:** Symptomatic and radiological success and complications for each study included in the systematic review

First author and publication year	Symptomatic success		Radiological success		Complications (*n*)	
	LD (%)	AS (%)	LD	AS	LD	AS
Agarwal et al. 2012	95	90	NA	NA	1 (infection)	
Bas et al. 2015	92.6	54.3	97.3%	60%	3 (1 hemorrhage; 2 adhesion)	
Choi et al. 2020	95	85	97.5%	60%	3 (1 fever; 1 ileus; 1 infection)	1 (fever)
Efesoy et al. 2015	97.6	94.7	95.2%	63.2%	1 (hemorrhage)	2 (fever)
Okeke et al. 2003	100	50	NA	NA	1 (hemorrhage)	
Shao et al. 2013	95.2	91.0	NA	NA	3 (post-operative complications)	

*AS* aspiration with sclerotherapy, *LD* laparoscopic deroofing, *NA* not available

### Complications, treatment duration, and post-treatment hospital stay

Included studies also reported information on complications and one of them explicitly declared to use the Clavien–Dindo Grading System [18]. The proportion of complications for each study and in each arm is reported in Table 3. Specifically, Agarwal and colleagues recorded 1 case of infection among 20 patients treated with laparoscopic deroofing [13]. Bas and colleagues reported that, for 3 patients treated with retroperitoneal laparoscopy, it was necessary to convert the operation to open surgery due to excessive bleeding or adhesions [16]. Choi and colleagues showed 3 patients with complications in the laparoscopic group (i.e., fever, ileus, or infected wound), while 1 patient who underwent aspiration with sclerotherapy had the fever [1]. Efesoy and colleagues reported 2 cases of fever among patients treated with aspiration and sclerotherapy and 1 case of hemorrhage among those who underwent laparoscopic deroofing [15]. Similarly, Okeke and colleagues also recorded 1 case of hemorrhage in the laparoscopic deroofing arm [17]. The higher proportion of complications in the latter group was also confirmed by Shao and colleagues, with 3 patients who presented post-operative complications [18]. Beyond a lower rate of complications, aspiration with sclerotherapy also exhibited a shorter procedural time, as reported by 4 studies included in the present systematic review [1, 13, 15, 18]. Specifically, the average duration of the intervention ranged from 30 to 49 min for aspiration with sclerotherapy, and from 59 to 112 min for laparoscopic deroofing. Moreover, also post-treatment hospital stay was shorter in the aspiration with sclerotherapy group, ranging from 2 h to 3 days. By contrast, patients who underwent laparoscopic deroofing stayed in hospital from 1 to 6 days. Finally, 3 studies [1, 15, 18] also estimated total costs associated with each treatment, although it was not clear which procedures were considered for this estimation. Thus, even here, aspiration with sclerotherapy was better than laparoscopic deroofing, with a mean total cost ranging from to 125 USD to 1256 USD. The laparoscopic approach was instead more expensive with a total cost of 729–2343 USD.

### Quality assessment

According to the criteria from the OCEBM, the overall level of evidence was rated as level 2. With respect to potential bias for non-randomized studies, all studies were considered as at low or moderate risks with slight differences across different domains (Table S2).

## Discussion

Symptoms and complications related to simple renal cysts, though not frequent, require a non-conservative approach that in the current state, however, is not guided by available indications or guidelines for their management. In 2018, Eissa and colleagues summarized existing evidence on the non-conservative management of simple renal cysts in adults, in a narrative review of more than 40 articles [5]. The authors considered all types of intervention against simple renal cysts and concluded that different factors influenced the choice of which treatment was more appropriated, including natural history of cysts, presence and type of symptoms and complications, and eventually patients’ opinion [5]. While open surgery is now considered an historical procedure that is no longer performed, simple aspiration is much less invasive, but it is also characterized by high recurrence rate [5]. For this reason, combining aspiration with sclerotherapy is recommended to prevent the re-accumulation of fluid after aspiration [19]. Indeed, aspiration with sclerotherapy is one of the most common minimally invasive procedures for the management of simple renal cysts, especially indicated for small and medium cysts [5]. On the other hand, however, laparoscopic deroofing is generally indicated for cysts with diameter > 10 cm, younger patients, and after failure of aspiration with sclerotherapy [20]. Yet, some authors also recommended the use of laparoscopic deroofing for cysts larger than 6 cm [3, 4]. Accordingly, there is a partial overlap between aspiration with sclerotherapy and laparoscopic deroofing, especially for the treatment of symptomatic simple renal cysts.

In this scenario, we carried out a systematic review and meta-analysis of studies comparing these two techniques in terms of radiological and symptomatic success. In line with previous evidence, our findings confirmed that laparoscopic deroofing had a higher success rate than aspiration with sclerotherapy, almost comparable with that of open surgery (i.e., more than 90%). Indeed, the risk of failure after aspiration and sclerotherapy was ~ 8 times higher for the radiological assessment and ~ 3 times higher for symptomatic assessment. On the contrary, however, aspiration with sclerotherapy was associated with less frequent complications, shorter treatment duration and post-treatment hospital stay, and lower costs. Thus, each option has its pros and cons, and further studies should be encouraged to understand whether patients’ characteristics, as well as cysts’ size and location, might tip the balance in favor of one of these treatments.

In fact, the limited number of studies, their low sample sizes, and the absence of data by patients’ characteristics, cysts’ size, and location hindered a stratified analysis which would have solved this issue. Moreover, there were other potentially influencing factors that should have been further evaluated, such as difference between transperitoneal and retroperitoneal approach for laparoscopy, and sclerosing agent, duration and number of sessions, patient position, and drainage for aspiration with sclerotherapy. Similarly, differences in the assessment of the efficacy of each treatment—especially in terms of radiological success—should have been considered. For instance, in Efesoy et al., a decrease of more than 50% in cyst’s size was considered as radiological success [15], while both Choi et al. and Bas et al. defined it as the absence of any cysts on ultrasonography or computed tomography [1, 16]. Finally, it should be considered that—though a low-to-moderate risk of bias—only Agarwal and colleagues presented results from a randomized prospective study [13].

With these considerations in mind, the present systematic review and meta-analysis point out benefits and drawbacks of the two most commonly used treatments against symptomatic simple renal cysts. However, they also underline the need for further randomized studies with larger sample size, more accurate information, and standardized protocols.

## Supplementary Information

Below is the link to the electronic supplementary material.

Supplementary file 1 (DOC 71 KB)

## Data Availability

Data and materials are available from the corresponding authors upon reasonable request.
